# Gender differences in disability after sickness absence with musculoskeletal disorders: five-year prospective study of 37,942 women and 26,307 men

**DOI:** 10.1186/1471-2474-12-37

**Published:** 2011-02-07

**Authors:** Sturla Gjesdal, Espen Bratberg, John G Mæland

**Affiliations:** 1 Department of Public Health and Primary Health Care, University of Bergen, Bergen, Norway; 2 Program for Health Economics, University of Bergen, Bergen, Norway; 3 Department of Economics, University of Bergen, Bergen, Norway

## Abstract

**Background:**

Gender differences in the prevalence and occupational consequences of musculoskeletal disorders (MSDs) are consistently found in epidemiological studies. The study investigated whether gender differences also exist with respect to chronicity, measured as the rate of transition from sickness absence into permanent disability pension (DP).

**Methods:**

Prospective national cohort study in Norway including all cases with a spell of sickness absence > eight weeks during 1997 certified with a MSD, 37,942 women and 26,307 men. The cohort was followed-up for five years with chronicity measured as granting of DP as the endpoint. The effect of gender was estimated in the full sample adjusting for sociodemographic factors and diagnostic distribution. Gender specific analyses were performed with the same explanatory variables. Finally, the gender difference was estimated for nine diagnostic subgroups.

**Results:**

The crude rate of DP was 22% for women and 18% for men. After adjusting for all sociodemographic variables, a slightly higher female risk of DP remained. However, additional adjustment for diagnostic distribution removed the gender difference completely. Having children and working full time decreased the DP risk for both genders, whereas low socioeconomic status increased the risk similarly. There was a different age effect as more women obtained a DP below the age of 50. Increased female risk of chronicity remained for myalgia/fibromyalgia, back disorders and "other/unspecified" after relevant adjustments, whereas men with neck disorders were at higher risk of chronicity.

**Conclusions:**

Women with MSDs had a moderately increased risk of chronicity compared to men, when including MSDs with a traumatic background. Possible explanations are lower income, a higher proportion belonging to diagnostic subgroups with poor prognosis, and a younger age of chronicity among women. When all sociodemographic and diagnostic variables were adjusted for, no gender difference remained, except for some diagnostic subgroups.

## Background

Musculoskeletal disorders (MSDs) affect a large proportion of the working population and their quality of life and contribute to increasing healthcare costs, lost work days and higher social insurance expenditures in most welfare states. One of the most worrying aspects of this "epidemic" is the high number of patients in working age that become permanently disabled and dependent of social insurance benefits because of MSD [1-7]. A growing literature has identified prognostic factors or causes of chronicity among patients with short-term disability caused by back pain [8-10] and other MSDs [11-15] in order to improve treatment and secondary prevention.

Indicators relevant to the epidemiology of MSD are scarce [1]. However, studies of sickness absence (SA) and permanent work-disability might outline the functional consequences of MSDs when cause-specific information is present [16]. Within universal social insurance systems, like in Sweden and Norway, population-based studies are feasible, since most women have paid work [7,17-20].

Gender differences are a key feature of the MSD epidemiology. A female excess in SA is found in countries with different social insurance systems and different levels of sickness absenteeism [7,17-19], even though some studies differ [21]. This corresponds to findings from epidemiological surveys of MSDs, in the general population [22,23] or in occupational samples [24,25] which have consistently found a higher prevalence among women [26]. A gender and site specific analysis of data from the Dutch cross sectional DMC3 study [22] showed a "slightly different" pattern in women and men with regard to the association of musculoskeletal pain with age, education, use of health care, disability and work-leave variables [27]. Behavioural factors and hazardous workplaces lead to more fractures and other injuries among men. Several studies on predictors of chronicity in MSD have shown that women with MSDs might have an increased risk of chronicity [13,15,17]. However, this has not been a subject for in-depth assessment.

Recently, investigators from different countries have discussed the need for gender specific studies of work related MSDs, without specifically mentioning the aspect of chronicity [28-30]. A main issue here is how statistical analyses should be performed, with adjustment or stratification for gender. Data from two large epidemiological studies from the US [29] and Canada [30] were analyzed both with adjustment for gender and separately for women and men in order to identify the full range of associations. Thus, in this study we decided to assess the gender difference with both approaches.

MSDs dominate social insurance expenditure in the Nordic countries, with respect to both short-term and permanent work disability. In the period 1994-2003, 150,374 women and 129,612 men obtained disability pension (DP) in Norway. Forty-one percent of these women and 28% of the men were certified with a MSD [31] whereas the incidence of DP caused by non-MSD was nearly similar. Thus, the higher prevalence of MSD among women has led to a "gender-gap" in the use of social insurance.

MSD might have different meanings and consequences for women and men and there are several explanations of the gender difference in MSD, with varying degrees of evidence [32] which may also have relevance with respect to chronicity: The exposure-theory claims that women generally have poorer working conditions, whereas the vulnerability theory claims that women are more strongly affected by pathogenic factors. Biological factors might explain the increased vulnerability and a higher prevalence of MSDs like osteoporosis, rheumatoid arthritis and fibromyalgia in women [33,34]. Mental/physical co-morbidity [35] and the interplay between pain and depression contribute to the functional consequences of MSDs, since depression is more common in women [36] and may affect pain differently [37] There are also differences in health beliefs and help seeking [27,38,39]. Gender differences in self rated health has been shown to vary in countries with different welfare state models, with the largest female/male difference in the Nordic countries [40]. The double-burden theory implies that the combined exposure to stressors in the family and at work might lead to musculoskeletal problems and SA [41,42] which might have different consequences depending on the extent of gender inequality [43,44]. Finally, socioeconomic variables play an important role in musculoskeletal morbidity [17,18,23,45,46] and different positions in society and workplace hierarchies might therefore also contribute to gender differences. Low socioeconomic status, education and income are both exposures and might lead to increased vulnerability. Adjusting for socioeconomic variables might reduce the gender differences significantly [17,18]. When also including workplace factors, the gender difference might "disappear" completely [21].

The aims of this national prospective cohort study were: To assess the chronicity/transition into permanent DP after sickness absence with a MSD and to investigate possible gender differences. If a gender difference is present, to investigate possible socioeconomic and medical (biological) explanations.

## Methods

### Design

Register-based, national, prospective cohort study.

### The origin of data and ethical considerations

The study was approved by the Norwegian Data Inspectorate. Explanatory variables originated from public registers merged by means of the national ID number. The data were obtained from an anonymous research database (FDtrygd) established by Statistics Norway and the National Insurance Services (NIS).

### Population at risk, inclusion criteria and participants

On January 1 1997, 1,019,216 men and 920,139 women aged 16-62 years were vocationally active and covered by the national SA benefit scheme in Norway. More than 70% of sickness certificates are issued by GPs, and the International Classification of Primary Health Care (ICPC) has been used by NIS since 1990 [47]. ICPC is organised in chapters corresponding to organ systems. Chapter L includes MSD and has a total of 53 sub-diagnoses: L01-L29 indicate symptoms like "back pain" and L70-L99 disorders or syndromes [48]. The ICPC L-chapter also includes MSDs with a traumatic background like fractures, sprains and other soft tissue injuries. All cases with a spell of SA > eight weeks, starting in 1997 with a main sick-leave diagnosis indicating a MSD were included, a total of 37,942 women and 26,307 men. This corresponds to an annual incidence of 4.1/100 for vocationally active women and 2.5/100 for men. Table 1 shows the most frequent MSD diagnoses. Sick-leave diagnoses were organised into nine groups based on localisation or pathophysiology: back disorders, neck disorders, non-traumatic disorders in the upper extremities, non-traumatic disorders in the lower extremities, fractures and other injuries, muscular pain/fibromyalgia, rheumatoid arthritis and similar disorders, osteoarthrosis, and "other". Back problems were the largest group affecting 29% of the women and 34% of the men on SA, an annual incidence of 1.2/100 and 0.9/100 respectively.

**Table 1:** The most frequent musculoskeletal diagnoses in sick leave >8 weeks in Norway in 1997, according to The International Classification of Primary Health Care (ICPC): N and percent of musculoskeletal cases, and percent obtaining a disability pension (DP)

ICPC		Men				Women		
Code	Diagnosis	N	%	%DP		N	%	%DP
L84	Back syndrome without radiation	3579	13.6	17.2		5165	13.6	19.1
L86	Back syndrome with radiation	3979	15.1	18.4		3607	9.5	23.0
L83	Neck syndrome	2005	7.6	25.0		4340	11.4	23.9
L92	Shoulder syndrom	2179	8.3	19.6		3781	10.0	22.7
L99	Musculoskeletal disorder NUD	913	3.5	19.2		4780	12.6	12.4
L93	Epicondylitis/tendinits forearm	1699	6.5	14.7		2652	7.0	20.2
L02	Back symptoms	1042	4.0	17.5		1857	4.9	17.2
L88	Rheumatoid arthritis	777	3.0	39.4		1220	3.2	50.3
L76	Other fractures	1009	3.8	13.3		613	1.6	17.8
L73	Fracture ankle/leg	803	3.1	4.7		630	1.7	11.4
L18	Widespread muscular pain	144	0.5	28.5		957	2.5	49.0
L01	Neck symptoms	330	1.3	21.8		676	1.8	23.1
L96	Acute inner injury of knee	632	2.4	8.2		333	0.9	14.1
L72	Fracture forearm	349	1.3	6.9		536	1.4	15.7
L08	Shoulder symptoms	332	1.3	19.3		406	1.1	19.2
L89	Osteoarthrosis of the hip	250	1.0	41.6		406	1.1	48.3

### Follow-up and endpoint

The study cohort was followed from the start of the index spell until December 31, 2002. The maximum and mean follow-up period was 83 and 61 months respectively. The endpoint was the date of granting a DP, obtained from the DP register, which is complete since this is the basis for payment of pensions.

### Explanatory variables and measurements

Diagnostic group, age, gender, annual income, educational attainment (years), weekly working hours, and whether living with children, were used as explanatory variables, shown in table 2. Those with no missing variables, 28,705 women and 19,837 men, were included in the statistical analyses.

**Table 2:** Frequencies of explanatory variables N (%), and the proportions N (%) obtaining disability pension (DP) during 5 years follow-up according to musculoskeletal groups and sociodemographic variables

	Men				Women			
Explanatory variables	N	%	DP	DP%	N	%	DP	DP%
Rheumatoid arthritis	777	3.0	306	39.4	1220	3.2	614	50.3
Osteoarthrosis	644	2.4	269	41.8	880	2.3	452	51.4
Myalgia/fibromyalgia	523	2.0	145	27.7	1836	4.8	757	41.2
Neck problems	2335	8.9	574	24.6	5016	13.2	1194	23.8
Back problems	8979	34.1	1598	17.8	11164	29.4	2246	20.1
Other	1829	7.0	288	15.7	5838	15.4	812	13.9
Upper extremities, non traumatic	4625	17.6	792	17.1	7300	19.2	1565	21.4
Lower extremities, non traumatic	874	3.3	120	13.7	1053	2.8	218	20.7
Fractures and injuries	5721	21.7	510	8.9	3635	9.6	551	15.2
**All**	**26307**	**100.0**	**4602**	**17.5**	**37942**	**100.0**	**8409**	**22.2**
**Age**								
16-29	5122	19,5	98	1.9	7448	19.6	163	2.2
30-39	7698	29,3	516	6.7	9992	26.3	886	8.9
40-49	6989	26,6	1162	16.6	10183	26.8	2418	23.7
50-59	5622	21,4	2312	41.1	9122	24.0	4229	46.4
60-62	876	3,3	514	58.7	1197	3.2	713	59.6
**Education (years)**								
Basic 7-9	3134	11.9	1311	41.8	4538	12.0	2238	49.3
Lower middle 10-12	9579	36.4	1534	16.0	14876	39.2	3625	24.4
Higher middle 13-15	10866	41.3	1393	12.8	12760	33.6	1764	13.8
Academic > 15	1479	5.6	182	12.3	4646	12.2	584	12.6
Missing	1249	4.7	182	14.6	1122	3.0	198	17.6
Annual income NOK*								
< 125,000	3163	12.0	479	15.1	9530	25.1	2131	22.4
125, 000-199,999	7083	26.9	1303	18.4	18615	49.1	4271	22.9
200,000-274 999	11954	45.4	2240	18.7	8640	22.8	1831	21.2
> 275,000	4048	15.4	580	14.3	1126	3.0	176	15.6
**Weekly working hours**								
0-19	835	3.2	220	26.3	5580	14.7	1535	27.5
20-29	565	2.1	101	17.9	6278	16.5	1389	22.1
30+	19328	73.5	2712	14.0	17648	46.5	3188	18.1
Missing	5579	21.2	1569	28.1	8436	22.2	2297	27.2

N = 26,307 men and 37,942 women sickness absent > 8 weeks

### The Norwegian social insurance system

All vocationally active residents are covered by the *SA benefit program*. A sickness certificate is required after three days' absence. After eight weeks, the certifying physician must complete a report including a diagnosis and plans for treatment. Therefore, eight weeks are considered as the start of LTSA. All inhabitants aged 18-66, are entitled to DP in case of permanent work disability caused by disease or injury. The DP program is thus equally accessible for both genders, even though employment rate is lower among women, and 40% work part time. People without work can obtain DP, but most DPs are granted after a process starting with SA benefits.

### Statistical analysis

Survival analysis was carried out. Diagnoses and sociodemographic factors were treated as categorical, and tested as predictors for obtaining a DP, by means of Cox proportional hazards analysis.

The hazards ratio (HR) with 95% confidence intervals (95% CI) was first estimated in the full sample with gender, age, education and income as explanatory variables. Then weekly working hours and family status (children <18 years) were included as covariates and finally diagnostic subgroup was included in a full model (table 3).

**Table 3:** Risk* of disability pension according to female gender, adjusted for sociodemographic and diagnostic variables

	Model 1			Model 2			Model 3		
Variables	HR	95%	CI	HR	95%	CI	HR	95%	CI
**Female gender**, ref men	1.21	1.15	1.27	1.09	1.03	1.14	1.03	0.98	1.09
**Age **ref <40 years									
40-49	4.04	3.74	4.35	3.97	3.68	4.29	3.87	3.59	4.18
50-59	9.33	8.67	10.03	8.38	7.77	9.05	8.21	7.60	8.86
60-62	13.02	11.79	14.38	11.21	10.12	12.42	10.91	9.84	12.10
**Education **ref >15 years									
< = 9 years	1.67	1.52	1.84	1.63	1.48	1.79	1.65	1.51	1.82
10-12 years	1.41	1.29	1.54	1.38	1.27	1.51	1.42	1.30	1.55
12-15 years	1.22	1.12	1.34	1.19	1.09	1.30	1.21	1.10	1.32
**Annual income **Ref >275 000 NOK									
< 124 999	1.61	1.45	1.78	1.18	1.06	1.32	1.24	1.11	1.38
125 000-199 999	1.75	1.60	1.92	1.63	1.49	1.79	1.63	1.49	1.79
200 000-274 999	1.51	1.38	1.65	1.51	1.38	1.64	1.49	1.36	1.62
**Weekly working hours **ref full time									
1-19				2.00	1.88	2.14	1.94	1.82	2.07
20-30				1.20	1.13	1.28	1.20	1.13	1.28
**Caring for children**									
No children				1.24	1.18	1.31	1.26	1.19	1.33
**Diagnosis **Ref back disorders									
Rheumatic disease							2.14	1.96	2.33
Osteoarthrosis							1.40	1.26	1.54
Myalgia/fibromyalgia							1.73	1.58	1.89
Neck problems							1.09	1.02	1.17
Other							0.89	0.82	0.96
Extremities							0.77	0.72	0.81
Fractures/injuries							0.50	0.46	0.54

N = 26,307 men and 37,942 women sickness absent > 8 weeks with musculoskeletal, followed up for 5 years

*Hazards ratio [HR] with 95% confidence intervals [95% CI]

Model 1: Gender variable adjusted for age

Model 2: Do + adjusted for education, income, weekly working hours, caring for children

Model 3: Do + adjusted for diagnosis distribution

In order to explore the gender difference further, HRs with 95%CI were estimated separately for men and women, shown in table 4, corresponding to the last model of table 3.

**Table 4:** Risk* of disability pension according to sociodemographic and diagnostic predictors separately for men and women

	Men				Women		
Variables	HR	95%	CI		HR	95%	CI
**Age ref < 40 years**							
40-49	4.15	3.64	4.73		3.76	3.42	4.13
50-59	10.35	9.11	11.75		7.34	6.65	8.09
60-62	15.29	12.91	18.10		9.14	8.00	10.43
**Education ref: > 15 years**							
< = 9 years	1.82	1.51	2.20		1.67	1.49	1.87
10-12 years	1.59	1.33	1.92		1.42	1.28	1.57
12-15 years	1.36	1.13	1.63		1.20	1.08	1.33
**Annual income (NOK) Ref >275 000**							
< 124 999	1.39	1.15	1.69		1.12	0.94	1.33
125 000-199 999	1.73	1.53	1.94		1.45	1.23	1.70
200 000-274 999	1.52	1.36	1.68		1.34	1.14	1.58
**Weekly working hrs, ref full time**							
1-19	2.86	2.47	3.31		1.82	1.69	1.95
20-30	1.80	1.46	2.22		1.15	1.08	1.24
**Caring for children**							
No children	1.20	1.10	1.31		1.30	1.21	1.39
**Diagnosis, ref back disorders**							
Rheumatic disease	2.08	1.78	2.43		2.16	1.94	2.40
Osteoarthrosis	1.45	1.23	1.71		1.36	1.20	1.54
Myalgia/fibromyalgia	1.29	1.03	1.61		1.78	1.61	1.97
Neck problems	1.29	1.14	1.45		1.01	0.93	1.10
Other	0.88	0.75	1.03		0.88	0.80	0.96
Extremities	0.82	0.74	0.90		0.74	0.69	0.80
Fractures/injuries	0.55	0.49	0.62		0.47	0.42	0.53

N = 26,307 men and 37,942 women sickness absent > 8 weeks with musculoskeletal followed up for 5 years

* Hazards ratio [HR] with 95% confidence interval [95%CI]

Table 5 shows the gender indicator estimated in stratified models for each diagnostic subgroup, adjusted for all socioeconomic factors, corresponding to model 2 in table 3.

**Table 5:** Risk* of disability pension according to female gender in musculoskeletal subgroups, adjusted for sociodemographic variables

	Model 1					Model 2			
Diagnostic group	HR	95%	CI	P		HR	95%	CI	P
Full sample	1.34	1.28	1.40	0.00		1.09	1.03	1.14	0.01
Upper limb	1.17	1.07	1.23	0.01		1.05	0.95	1.16	0.23
Lower limb	1.23	0.99	1.54	0.07		1.23	0.94	1.59	0.13
Other,unspecified	1.41	1.19	1.67	0.00		1.21	1.00	1.47	0.05
Back problems	1.40	1.29	1.52	0.00		1.10	1.00	1.21	0.05
Neck problems	1.05	0.93	1.19	0.44		0.83	0.72	0.96	0.01
Myalgia/fibromyalgia	1.76	1.40	2.22	0.00		1.44	1.12	1.85	0.00
Osteoarthrosis	1.28	1.06	1.54	0.01		0.89	0.71	1.11	0.29
Rheumatoid arthritis	1.48	1.24	1.76	0.00		1.13	0.92	1.38	0.26

N = 26,307 men and 37,942 women sickness absent > 8 weeks followed up for 5 years

* Hazards ratio [HR] with 95% confidence interval [95% CI]

Model 1: female gender, adjusted for age

Model 2: female gender adjusted for age, education, income, weekly working hours, caring for children

## Results

The mean age was nearly similar in the male and female samples, 40.4 versus 40.9 years. The mean educational level was slightly higher for the women 11.7 versus 11.3 years. However, mean annual income was 215,000 NOK for men versus 162,000 for women. (EUR 1 approximately 8 NOK). During follow-up 22% of the women and 18% of the men obtained a DP. Crude DP rates increased with age, lower education and low income for both genders. Twelve percent of both the female and male sample had only basic education (7-9 years). Of these, 49% of the women and 42% of the men obtained a DP. The crude risk of DP was higher for women in all diagnostic groups, except for neck problems and "other". DP rates for the diagnostic subgroups varied from 14% (other) to 51% (osteoarthrosis) among women and from 9% (fractures and injuries) to 41% (osteoarthrosis) among men (table 2).

When the full sample was analyzed together the risk or HR for obtaining DP related to female gender was 1.34 (95% CI 1.28-1.40) when adjusted only for age. When income and education were included, the HR decreased to 1.21 (1.15-1.27), and significantly more when adjusted for part-time work and family obligations 1.09 (1.03-1.14). However, only after including the diagnostic distribution the gender difference was completely "removed". In this final model the risk of DP according to diagnostic subgroups is also presented after adjustment for all socio-demographic variables (table 3).

The gender-stratified analysis showed that age above 50 and very low income, were more strongly linked to the risk of DP among the men, when adjusted for all other variables. Higher education had a similar protective effect for both genders. In both genders, those working part time and those without children had an increased risk of DP. Among women, the prevalence of myalgia/fibromyalgia was 2.5 times higher than among men and the risk of DP for women with this diagnosis was even higher than among those with osteoarthrosis. Among men, neck problems carried a higher DP risk than back problems. Cases with fractures or other injuries, 22% of the male and 10% of the female sample had a significantly lower risk than all other groups for both genders (table 4).

In the diagnosis-specific analyses (table 5), women had a higher risk of DP in all diagnostic groups, except for lower limb and neck problems when adjusted only for age. In the fully adjusted model women with back problems, myalgia/fibromyalgia and "other" had significantly higher risks of DP than men with similar diagnoses. Men with neck problems had still an increased risk of DP, compared to women. No gender difference was present among cases with inflammatory rheumatism, osteoarthrosis and problems in upper and lower extremities.

## Discussion

### Main findings

The distribution of diagnoses according to gender and localisations found in this study corresponds to previous studies of MSDs [15,23-26]. Back pain and non-traumatic disorders in the upper extremities represented nearly half of the cases. Contrary to most previous studies fractures/injuries, OA and inflammatory rheumatism were also included as specific categories.

The starting point of the study was a 60% greater incidence of SA with MSD among women compared to men (37,942 women and 26,307 men). The rate of chronicity measured as DP during follow-up was also slightly higher among the female sample, 22.2% versus 17.5% among the men. This combined effect explains that DP with MSD was twice as frequent among women compared to men during 1997-2002 [31].

The income difference was substantial when comparing the male and female samples, and adjustment for income removed approximately one-third of the crude difference in chronicity. Another third was removed by adjustment for working hours and family status. Among women, a higher proportion had disorders with poor prognosis, whereas disorders with favourable prognoses, especially fractures/injuries were more frequent among men. When adjusting also for this difference, no gender effect remained.

In some important subgroups, especially back disorders, a higher female risk of DP was still present after adjusting for all variables.

When analysing men and women separately some differences in the effects of covariates were present: Among women, myalgia/fibromyalgia had a poorer prognosis than OA. The age effect was also different. Both men and women caring for children below 18 years had a lower risk of DP than comparable persons without children. Working part time increased the risk of DP. These findings seem to refute the "double burden theory".

### Strengths of the study

This is a population-based study of musculoskeletal impairments in the working population using medically verified diagnoses, with data on 37,942 women and 26,307. Contrary to most previous studies which have targeted regional disorders, most frequently back and upper extremities, the present study includes all MSDs. Self-reported data seldom include more than the localisation of a MSD or musculoskeletal pain, irrespective of causal or biomedical aspects. When included after eight weeks on SA, patients normally have seen their GP several times and usually additional information from radiological examinations and laboratory tests is present and sometimes even assessment by specialists. Objective sociodemographic information was available for nearly all cases. The follow-up period was considerable and there were no dropouts, since all cases of DP, death or emigration were recorded by the authorities, and linked to the research database. Compared to previous epidemiological studies, based on self-reported pain, the present design might represent a better diagnostic validity.

### Limitations

There are several weaknesses of register-based studies with data from sickness certifications. Compared to clinical studies, with relatively few participants, there is a complete lack of health data other than a "main diagnosis" on the certificate. This means that the importance of co-morbidity and multi-site MSD cannot be examined. The importance of mental distress or depression among patients with MSD has been increasingly ascertained [35], and might also influence the gender differences

In the present study also data on workplace factors, family income and occupations were missing. There was some missing data with respect to weekly working hours, which is important for adjustment for part-time work, because of low quality of the Norwegian employment register. However more than 70% of the cases had no missing data and was included in the statistical analyses. Analyses performed with all participants gave similar results.

Annual rates of SA vary in Norway. During recessions, SA tends to be low, explained by different attitudes and changing composition of the workforce [49]. SA reached a low level in 1993, subsequently increasing until 2000. The inclusion year (1997) was thus a year with intermediate rates of SA in Norway [31].

The validity of the ICPC diagnoses might be questioned. The ICPC was developed as a tool for research in primary health care [48], and Norwegian GPs have used this classification for more than 15 years [47]. However, no scientific investigation has compared the ICPC diagnoses with a gold standard. We believe that few Norwegian GPs use osteoarthrosis as a diagnosis without radiological findings, or RA without previous assessment by a rheumatologist. In the present study, the ICPC codes were collapsed into broad categories, which might improve the validity. The diagnostic groups used in this study are quite similar to the groups used in a recent study of sickness absentees in Madrid [15]. Only one diagnosis was available for each case.

Our outcome measure was the granting of a DP during 5 years follow-up. We know however, that a number of patients who are not able to work because of their MSD, with or without co-morbidity, will not have obtained a DP five years after the onset of the disease. In stead they have long-term unemployment benefits or social assistance. Whether this is more common among men or women is not known, but could influence the results of the study.

Finally, in our statistical models, all covariates were restricted to have the same effect for men and women. Models without interactions might conceal gender differences in the effects of individual predictors of transition to DP. Both medical and sociodemographic factors (like age) might have different impacts on the musculoskeletal health of women and men.

### Comparisons with previous research findings

Gender differences in musculoskeletal morbidity and sickness absence have been explained with partly contradictory theories [18,21,32-34]. The present study confirmed that socioeconomic variables [17,18,21,23,45,46] also explain the slightly higher risk of chronicity in women. The "double-burden" theory [17,27,41-44] was refuted since no adverse effect of living in a family with children was observed. The DP risk related to working part time [17] was confirmed. Since shorter working hours will hardly increase the risk of DP, this finding is probably a result of selection effects; persons with health problems often decide to work part time only. Since diagnostic information was available, possible biological explanations were also assessed. A higher incidence of MSDs with poorer prognosis, like myalgia/fibromyalgia, and lower incidence of injuries increases the rate of chronicity among women.

## Conclusions

A higher prevalence of musculoskeletal morbidity, impairments and rates of sickness absence among women has been consistently found previously. This was also the starting point of this study, with nearly 60% more women in the study cohort. The same gender difference, however very moderate, was found with respect to chronicity, measured as the rate of transition into a permanent DP status, before any adjustments. The study showed, however, that this difference is present only in a few MSD-subgroups and is largely explained by socioeconomic variables. In the full sample no gender difference in chronicity remained after adjustment for all included confounders.

There is now considerable research activity into risk factors of permanent disability among individuals with MSDs [8-15,17]. Gender aspects should be included in this research. A future strategy to reduce the incidence and consequences of MSDs should include better protection of female workers and probably their general position in society. Rehabilitation programs for work-disabled workers should probably also have a gender specific design, even though gender differences in chronicity are small.

## Competing interests

The authors declare that they have no competing interests.

## Authors' contributions

SG, EB and JGM all participated in the conception and planning of the study, acquisition of data, data analysis and drafting of the manuscript which was finally accepted by all authors. SG was the main researcher in this project.

## Authors' information

SG is a general practitioner and professor at Department of Public health and Primary health care at The University of Bergen. His research interests include studies of the health among receivers of social insurance benefits and musculoskeletal epidemiology. EB is a professor in economics with a long career as a researcher within welfare state and social insurance economics and econometrics. He is at present the head of the Department of Economics at The University of Bergen. JGM is a professor and teacher at the Department of Public health and Primary health care at The University of Bergen and former director of the Research Centre of Health Promotion at The University of Bergen. His research interests are within social medicine and social inequalities of health. He is also a medical advisor for The Social Insurance Services

## Pre-publication history

The pre-publication history for this paper can be accessed here:

http://www.biomedcentral.com/1471-2474/12/37/prepub

## References

[B1] Woolf AD Akesson K Understanding the burden of musculoskeletal conditions. The burden is huge and not reflected in national health priorities Bmj 2001 322 1079 80 10.1136/bmj.322.7294.1079 11337425 PMC1120225

[B2] Smolen JS Combating the burden of musculoskeletal conditions Ann Rheum Dis 2004 63 329 10.1136/ard.2004.022137 15020321 PMC1754933

[B3] Armstrong R Wilkie R Musculoskeletal problems and work in the UK--time for a new approach? Rheumatology 2009 48 709 10 10.1093/rheumatology/kep071 19364766

[B4] Mantyselka PT Kumpusalo EA Ahonen RS Takala JK Direct and indirect costs of managing patients with musculoskeletal pain-challenge for health care Eur J Pain 2002 6 141 148 10.1053/eujp.2001.0311 11900474

[B5] Yelin E Cost of musculoskeletal diseases: impact of work disability and functional decline J Rheumatol Suppl 2003 68 8 11 14712615

[B6] Isenberg DA 30 million, around 10 000 ... and 18 ... figuring out the optimal treatment for musculoskeletal conditions in the National Health Service Rheumatology 2007 46 1219 20 10.1093/rheumatology/kem083 17569748

[B7] Hansson T Jensen I Swedish Council on Technology Assessment in Health Care. Sickness absence due to back and neck disorders Scand J Public Health Suppl 2004 63 109 51 10.1080/14034950410021862 15513655

[B8] Croft PR Macfarlane GJ Papageorgiou AC Thomas E Silman AJ Outcome of low back pain in general practice: a prospective study Bmj 1998 316 1356 1359 9563990 10.1136/bmj.316.7141.1356 PMC28536

[B9] Cassidy JD Cote P Carroll LJ Kristman V Incidence and course of low back pain episodes in the general population Spine 2005 30 2817 2823 10.1097/01.brs.0000190448.69091.53 16371911

[B10] Pincus T Santos R Breen A Burton AK Underwood M A review and proposal for a core set of factors for prospective cohorts in low back pain: a consensus statement Arthritis Rheum 2008 59 14 24 10.1002/art.23251 18163411

[B11] Turner JA Franklin G Fulton-Kehoe D Egan K Wickizer TM Lymp JF Sheppard L Kaufman JD Prediction of chronic disability in work-related musculoskeletal disorders: a prospective, population-based study BMC Musculoskeletal Disorders 2004 5 14 10.1186/1471-2474-5-14 15157280 PMC428578

[B12] Stover B Wickizer TM Zimmerman F Fulton-Kehoe D Franklin G Prognostic factors of long-term disability in a workers' compensation system J Occup Environ Med 2007 49 31 40 10.1097/01.jom.0000250491.37986.b6 17215711

[B13] Mallen CD Peat G Thomas E Dunn KM Croft PR Prognostic factors for musculoskeletal pain in primary care: a systematic review Br J Gen Pract 2007 57 655 661 17688762 PMC2099673

[B14] Lotters F Burdorf A Prognostic factors for duration of sickness absence due to musculoskeletal disorders Clin J Pain 2006 22 212 221 10.1097/01.ajp.0000154047.30155.72 16428958

[B15] Abasolo L Carmona L Lajas C Candelas G Blanco M Loza E Hernandez-Garcia C Jover JA Prognostic factors in short-term disability due to musculoskeletal disorders Arthritis Rheum 2008 59 489 496 10.1002/art.23537 18383421

[B16] Marmot M Feeney A Shipley M Sickness absence as a measure of health status and functioning: from the UK Whitehall II study J Epidemiol Community Health 1995 49 124 30 10.1136/jech.49.2.124 7798038 PMC1060095

[B17] Gjesdal S Bratberg E The role of gender in long-term sickness absence and transition to permanent disability benefits. Results from a multi-register based, prospective study in Norway 1990-1995 Eur J Public Health 2002 12 180 6 10.1093/eurpub/12.3.180 12232956

[B18] Brage S Nygard JF Tellnes G The gender gap in musculoskeletal-related long-term sickness absence in Norway Scand J Soc Med 1998 26 34 43 9526762 10.1177/14034948980260010901

[B19] Alexanderson K Leijon M Akerlind I Rydh H Bjurulf P Epidemiology of sickness absence in a Swedish county in 1985, 1986 and 1987. A three year longitudinal study with focus on gender, age and occupation Scand J Soc Med 1994 22 27 34 8029663 10.1177/140349489402200105

[B20] Hagen KB Tambs K Bjerkedal T A prospective cohort study of risk factors for disability retirement because of back pain in the general working populatiotion Spine 2002 27 1790 1796 10.1097/00007632-200208150-00019 12195073

[B21] Laaksonen M Martikainen P Rahkonen O Lahelma E Explanations for gender differences in sickness absence: evidence from middle-aged municipal employees from Finland Occup Environ Med 2008 65 325 30 10.1136/oem.2007.033910 18252767

[B22] Picavet HS Schouten JS Musculoskeletal pain in the Netherlands: prevalences, consequences and risk groups, the DMC(3)-study Pain 2003 102 167 78 10.1016/s0304-3959(02)00372-x 12620608

[B23] Urwin M Symmons D Allison T Brammah T Busby H Roxby M Simmons A Williams G Estimating the burden of musculoskeletal disorders in the community: the comparative prevalence of symptoms at different anatomical sites, and the relation to social deprivation Ann Rheum Dis 1998 57 649 655 10.1136/ard.57.11.649 9924205 PMC1752494

[B24] de Zwart BC Broersen JP Frings-Dresen MH van Dijk FJ Musculoskeletal complaints in The Netherlands in relation to age, gender and physically demanding work Int Arch Occup Environ Health 1997 70 352 360 10.1007/s004200050229 9352339

[B25] Leroux I Dionne CE Bourbonnais R Brisson C Prevalence of musculoskeletal pain and associated factors in the Quebec working population Int Arch Occup Environ Health 2005 78 379 386 10.1007/s00420-004-0578-2 15843954

[B26] Wijnhoven HA de Vet HC Picavet HS Prevalence of musculoskeletal disorders is systematically higher in women than in men Clin J Pain 2006 22 717 24 10.1097/01.ajp.0000210912.95664.53 16988568

[B27] Wijnhoven HA de Vet HC Picavet HS Sex differences in consequences of musculoskeletal pain Spine 2007 32 1360 7 10.1097/BRS.0b013e31805931fd 17515827

[B28] Artazcoz L Borrell C Cortes I Escriba-Aguir V Cascant L Occupational epidemiology and work related inequalities in health: a gender perspective for two complementary approaches to work and health research J Epidemiol Community Health 2007 61 Suppl 2 ii39 45 10.1136/jech.2007.059774 18000116 PMC2465767

[B29] Silverstein B Fan ZJ Smith CK Bao S Howard N Spielholz P Bonauto D Viikari-Juntura E Gender adjustment or stratification in discerning upper extremity musculoskeletal disorder risk? Scand J Work Environ Health 2009 35 2 113 126 19294319 10.5271/sjweh.1309

[B30] Messing K Tissot F Stock SR Should studies of risk factors for musculoskeletal disorders be stratified by gender? Lessons from the 1998 Quebec Health and Social Survey Scand J Work Environ Health 2009 35 96 112 19305934 10.5271/sjweh.1310

[B31] Norwegian Insurance Services Statistical Yearbooks of Social Insurance 1998-2003 Oslo: (NAV.no)

[B32] Wijnhoven HA de Vet HC Picavet HS Explaining sex differences in chronic musculoskeletal pain in a general population Pain 2006 146 158 66 10.1016/j.pain.2006.04.012 16716517

[B33] Fillingim RB Sex, gender, and pain: women and men really are different Curr Rev Pain 2000 4 24 30 10998712 10.1007/s11916-000-0006-6

[B34] Tosi LL Boyan BD Boskey AL Does sex matter in musculoskeletal health? The influence of sex and gender on musculoskeletal health J Bone Joint Surg Am 2005 87 1631 47 10.2106/JBJS.E.00218 15995134

[B35] Kessler RC Ormel J Demler O Stang PE Comorbid mental disorders account for the role impairment of commonly occurring chronic physical disorders: results from the National Comorbidity Survey J Occup Environ Med 2003 45 1257 1266 10.1097/01.jom.0000100000.70011.bb 14665811

[B36] Munce SE Stewart DE Gender differences in depression and chronic pain conditions in a national epidemiologic survey Psychosomatics 2007 48 94 9 10.1176/appi.psy.48.5.394 17878497

[B37] Keogh E McCracken LM Eccleston C Gender moderates the association between depression and disability in chronic pain patients Eur J Pain 2006 10 413 22 10.1016/j.ejpain.2005.05.007 16009583

[B38] Barsky AJ Peekna HM Borus JF Somatic symptom reporting in women and men J Gen Intern Med 2001 16 266 75 10.1046/j.1525-1497.2001.016004266.x 11318929 PMC1495200

[B39] Hooftman WE Westerman MJ van der Beek AJ Bongers PM van Mechelen W What makes men and women with musculoskeletal complaints decide they are too sick to work? Scand J Work Environ Health 2008 34 2 107 112 18470439 10.5271/sjweh.1221

[B40] Bambra C Pope D Swami V Stanistreet D Roskam A Kunst A Scott-Samuel A Gender, health inequalities and welfare state regimes: a cross-national study of 13 European countries J Epidemiol Community Health 2009 63 1 38 44 10.1136/jech.2007.070292 18768570

[B41] Artazcoz L Artieda L Borrell C Cortès I Benach J García V Combining job and family demands and being healthy: what are the differences between men and women? Eur J Public Health 2004 14 43 8 10.1093/eurpub/14.1.43 15080390

[B42] Vaananen A Kevin MV Ala-Mursula L Pentti J Kivimaki M Vahtera J The double burden of and negative spillover between paid and domestic work: associations with health among men and women Women Health 2004 40 1 18 15829442 10.1300/j013v40n03_01

[B43] Mastekaasa A Parenthood, gender and sickness absence Soc Sci Med 2000 50 1827 42 10.1016/S0277-9536(99)00420-7 10798335

[B44] Bratberg E Dahl S Risa A 'The double burden' Do combinations of Career and Family Obligations Increase Sickness Absence among Women? Eur Sociological Review 2002 18 233 249 10.1093/esr/18.2.233

[B45] Macfarlane GJ Norrie G Atherton K Power C Jones GT The influence of socio-economic status on the reporting of regional and widespread musculoskeletal pain: results from the 1958 British Birth Cohort Study Ann Rheum Dis 2009 68 1591 5 10.1136/ard.2008.093088 18952642

[B46] Gjesdal S Bratberg E Maeland JG Musculoskeletal impairments in the Norwegian working population: the prognostic role of diagnoses and socioeconomic status: a prospective study of sickness absence and transition to disability pension Spine 2009 34 1519 25.23 10.1097/BRS.0b013e3181a8dee3 19525845

[B47] Brage S Bentsen BG Bjerkedal T Nygard JF Tellnes G ICPC as a standard classification in Norway Fam Pract 1996 13 391 396 10.1093/fampra/13.4.391 8872099

[B48] Hofmans-Okkes IM Lamberts H The International Classification of Primary Care (ICPC): new applications in research and computer-based patient records in family practice Fam Pract 1996 13 294 302 10.1093/fampra/13.3.294 8671139

[B49] Askildsen JE Bratberg E Nilsen OA Unemployment, labor force composition and sickness absence: a panel data study Health Econ 2005 14 1087 101 10.1002/hec.994 15791654

